# Supplementary material for: Acute session of three endurance exercise intensities alters subcutaneous adipose tissue transcriptome in regular exercisers
Source: bioRxiv. 2025 May 8:2025.05.02.651890. Preprint. [Version 1] doi: 10.1101/2025.05.02.651890 (PMC12247987; doi:10.1101/2025.05.02.651890)
Supplement: Supplement 1 [file NIHPP2025.05.02.651890v1-supplement-1.pdf]

bioRxiv preprint doi: https://doi.org/10.1101/2025.05.02.651890; this version posted May 8, 2025. The copyright holder for this preprint (which was not certified by peer review) is the author/funder, who has granted bioRxiv a license to display the preprint in perpetuity. It is made available under aCC-BY 4.0 International license.

Figure S1A

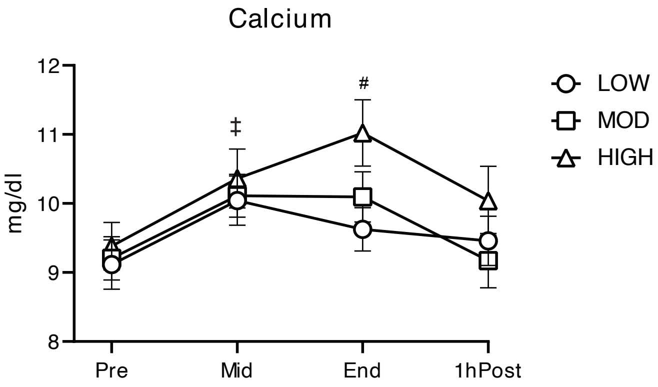

Figure S1B

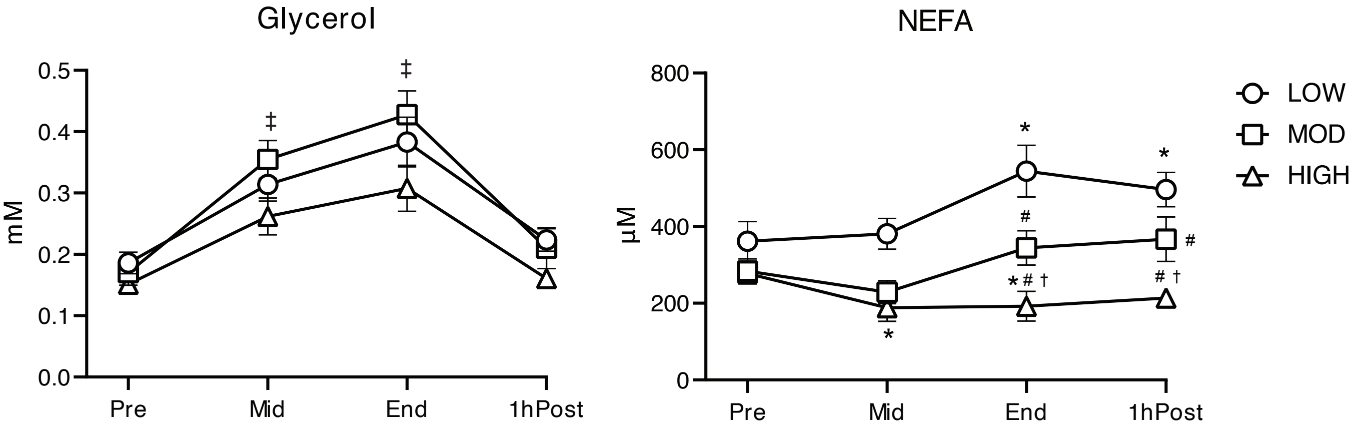

Figure S1C

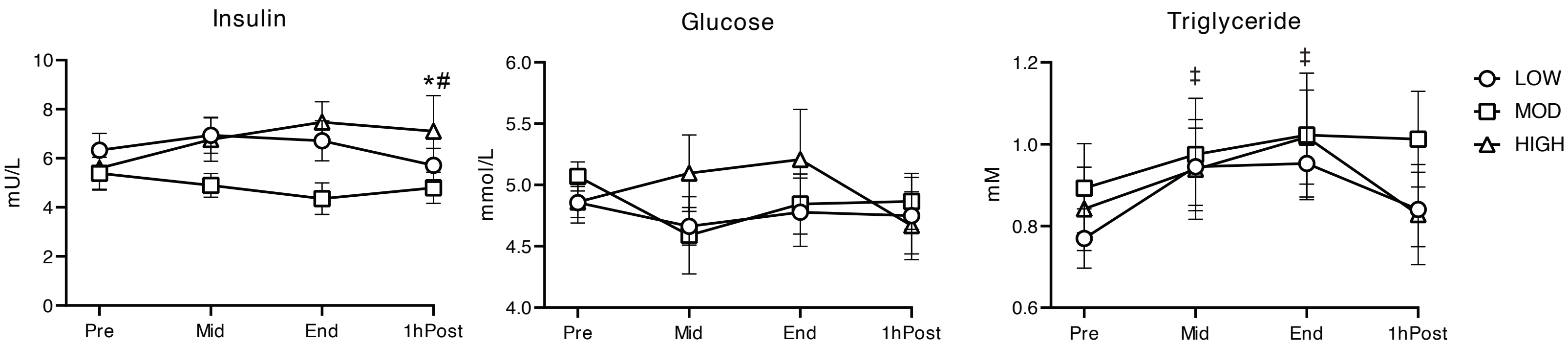

Figure S1. Concentrations of circulating calcium, glycerol, fatty acid, insulin, glucose, and triglyceride during and post LOW, MOD, and HIGH.

A) Plasma calcium concentration. B) Plasma glycerol and fatty acid concentrations. C) Plasma insulin, glucose,and triglyceride concentrations. ‡main effect of time ($p<0.05$). *$p<0.05$ vs. Pre; †$p<0.05$ vs. MOD; #$p<0.05$ vs. LOW. Significant overall Time x Group interaction effects were detected in fatty acid ($p<0.001$, Type III ANOVA). There was a trend of Time (i.e., End) x Group (i.e., HIGH) interaction effect for calcium ($p=0.06$). Post-hoc pairwise comparison suggested significant difference in calcium concentration between LOW vs. HIGH at End ($p=0.01$). There was a significant Time (i.e., 1hPost) x Group (i.e., HIGH) interaction effect for insulin ($p=0.025$). Data is presented as Mean±SEM. Detailed p-values are included in the Result.

bioRxiv preprint doi: https://doi.org/10.1101/2025.05.02.651890; this version posted May 8, 2025. The copyright holder for this preprint (which was not certified by peer review) is the author/funder, who has granted bioRxiv a license to display the preprint in perpetuity. It is made available under aCC-BY 4.0 International license.

Figure S2A

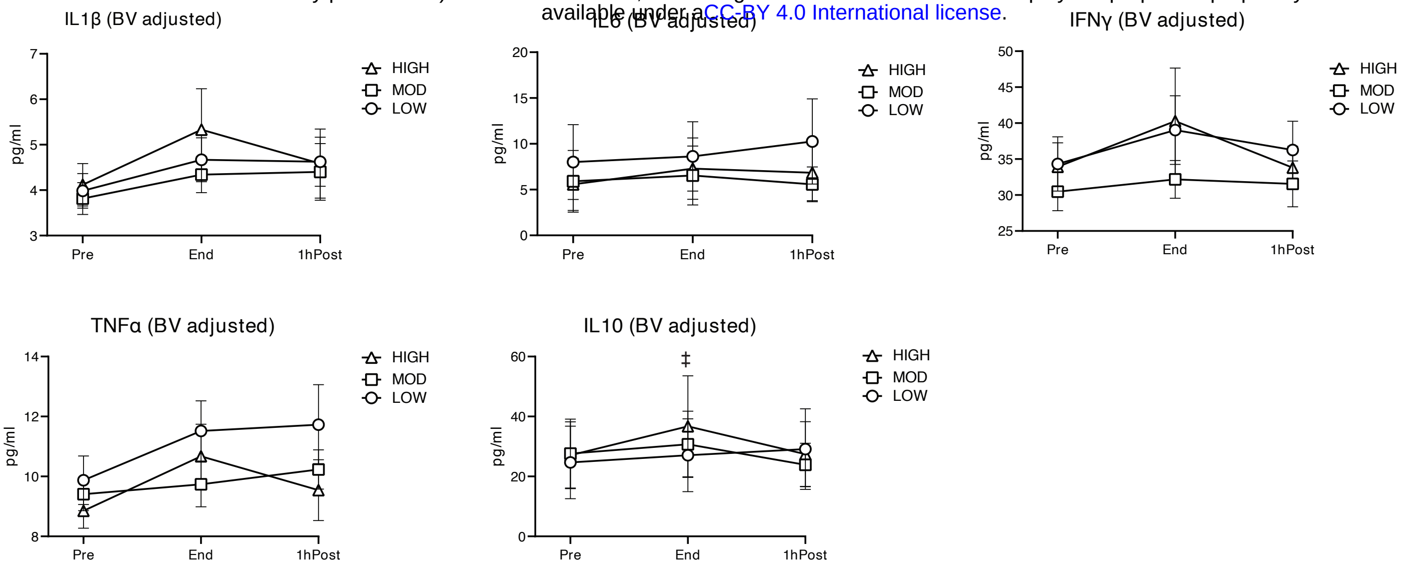

Figure S2B

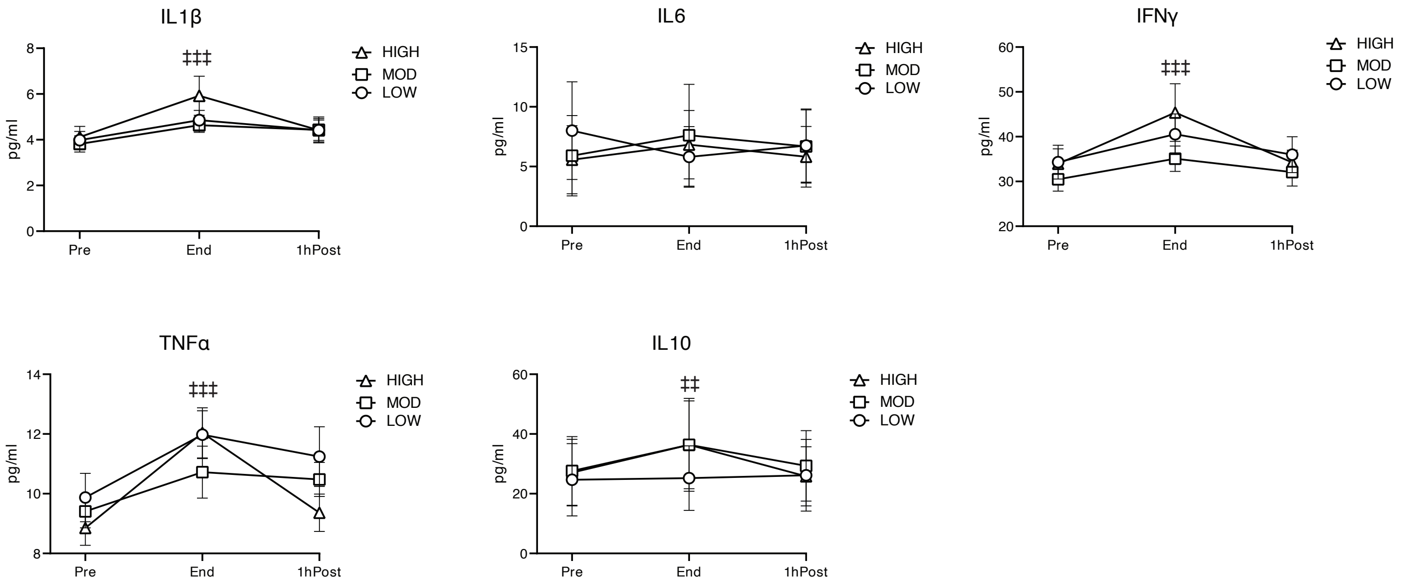

Figure S2. Concentrations of circulating cytokines during and post LOW, MOD, and HIGH.
A) Blood volume-adjusted plasma IL1β, IL6, IFNγ,TNFα, IL10 concentrations. B) Unadjusted plasma IL1β, IL6, IFNγ,TNF α, IL10 concentrations. ‡main effect of time ($p<0.05$); ‡‡main effect of time ($p<0.01$); ‡‡‡main effect of time ($p<0.001$).. Sample sizes for IL10 – LOW: n=14; MOD: n=15; HIGH: n=14. Sample sizes for IL6 – LOW: n=10; MOD: n=14; HIGH: n=13. Sample sizes for IL1β, IFNγ, and TNFα - LOW: n=15; MOD: n=15; HIGH: n=15. Data is presented as Mean±SEM. BV, Blood volume.

bioRxiv preprint doi: https://doi.org/10.1101/2025.05.02.651890; this version posted May 8, 2025. The copyright holder for this preprint (which was not certified by peer review) is the author/funder, who has granted bioRxiv a license to display the preprint in perpetuity. It is made available under aCC-BY 4.0 International license.

Fig S3A

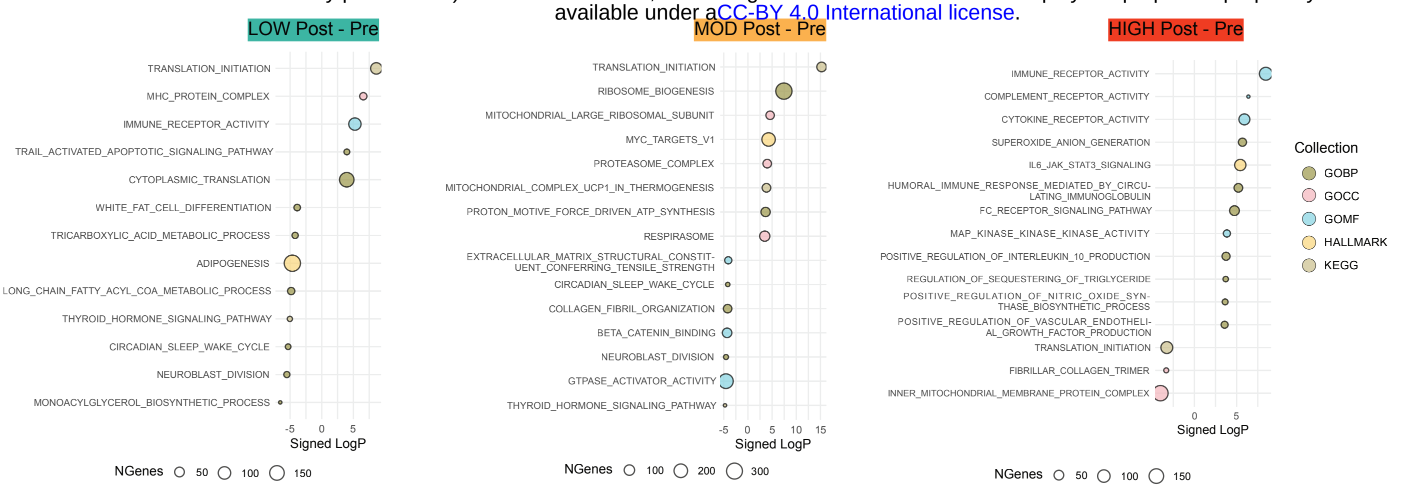

Fig S3B

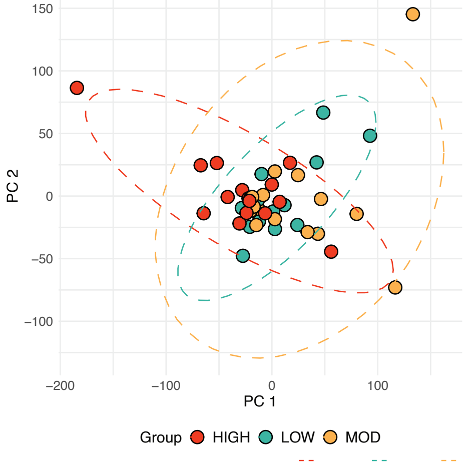

Fig S3C

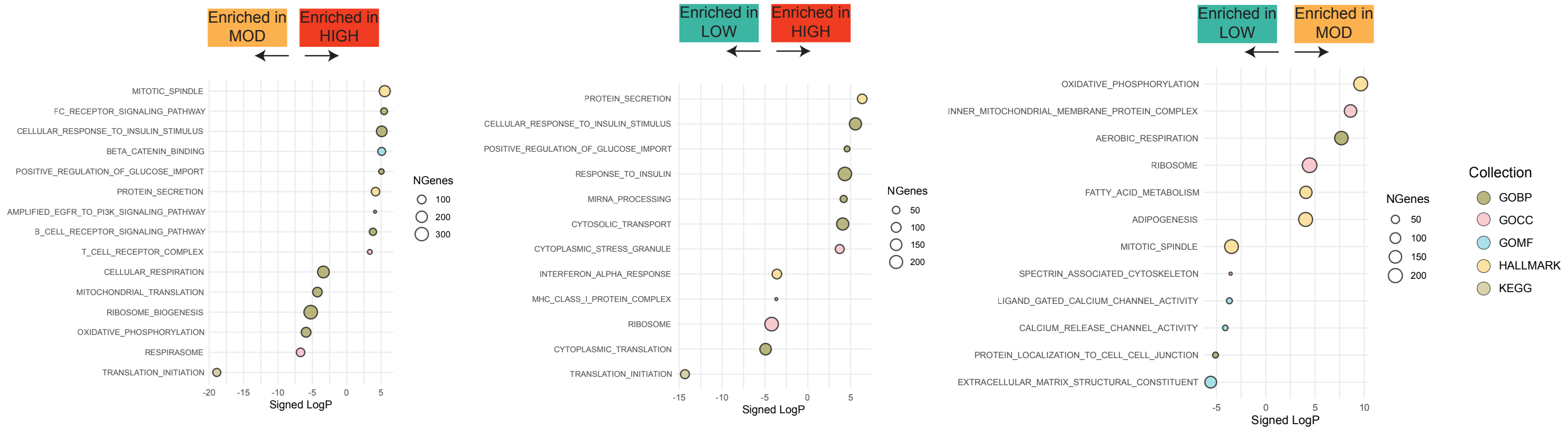

Figure S3. Gene set testing using CAMERA
A) Enriched biological pathways from each exercise treatment (comparing post- vs. pre-exercise), derived from CAMERA. Signed -log(p-value) from DESeq2 is used as the input for each gene. Positive values in the X-axis refers to upregulation after exercise. B) PCA of gene expression changes (delta of post/pre gene expression) in study subjects. Each point represents the subject. C) Enriched biological pathways from each exercise treatment (comparing exercise groups), derived from CAMERA. CAMERA, Competitive gene set test accounting for inter-gene correlation; PCA, Principal Component Analysis. Only significant terms (adjusted p<0.05) are shown in panel A and C.

bioRxiv preprint doi: https://doi.org/10.1101/2025.05.02.651890; this version posted May 8, 2025. The copyright holder for this preprint (which was not certified by peer review) is the author/funder, who has granted bioRxiv a license to display the preprint in perpetuity. It is made available under aCC-BY 4.0 International license.

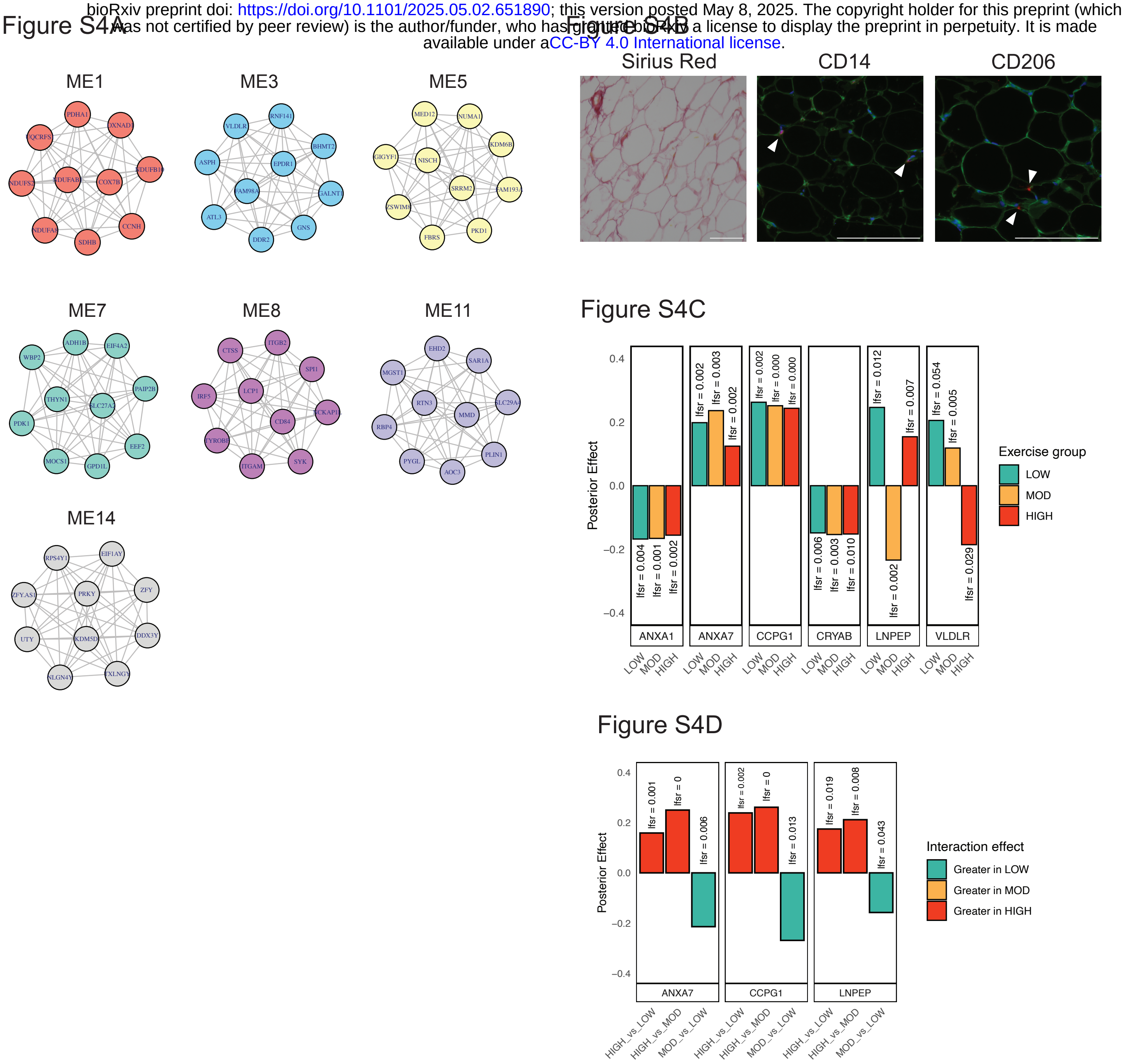

Figure S4. Integration of aSAT transcriptomics with clinical/tissue traits.
A) Top 10 hub-genes from selected modules. Grey edges indicate intercorrelation between genes. B) Representative images of aSAT histology. Sirius Red was used to stain collagen type I and III depositions. CD14 was used as a marker for pro-inflammatory macrophages. CD206 was used as a marker for anti-inflammatory macrophages. Positive stains of CD14 and CD206 are marked with white arrows. C) DEGs among top 20 hub-genes of module 3. D) DEGs among top 20 hub-genes of module 3 that had significant group x interaction effect; ANXA7, CCPG1, and LNPEP.
